# A Molecular Rotor that Measures Dynamic Changes of Lipid Bilayer Viscosity Caused by Oxidative Stress

**DOI:** 10.1002/chem.201601925

**Published:** 2016-08-03

**Authors:** Aurimas Vyšniauskas, Maryam Qurashi, Marina K. Kuimova

**Affiliations:** ^1^ Chemistry DepartmentImperial College LondonExhibition RoadLondonSW7 2AZUK

**Keywords:** fluorescent probes, lipid oxidation, liposomes, microviscosity, molecular rotors

## Abstract

Oxidation of cellular structures is typically an undesirable process that can be a hallmark of certain diseases. On the other hand, photooxidation is a necessary step of photodynamic therapy (PDT), a cancer treatment causing cell death upon light irradiation. Here, the effect of photooxidation on the microscopic viscosity of model lipid bilayers constructed of 1,2‐dioleoyl‐*sn*‐glycero‐3‐phosphocholine has been studied. A molecular rotor has been employed that displays a viscosity‐dependent fluorescence lifetime as a quantitative probe of the bilayer's viscosity. Thus, spatially‐resolved viscosity maps of lipid photooxidation in giant unilamellar vesicles (GUVs) were obtained, testing the effect of the positioning of the oxidant relative to the rotor in the bilayer. It was found that PDT has a strong impact on viscoelastic properties of lipid bilayers, which ‘travels’ through the bilayer to areas that have not been irradiated directly. A dramatic difference in viscoelastic properties of oxidized GUVs by Type I (electron transfer) and Type II (singlet oxygen‐based) photosensitisers was also detected.

## Introduction

Unsaturated lipids are commonly found in a variety of biological membranes and are vulnerable to reactive oxygen species (ROS) such as singlet oxygen and oxygen‐based radicals. Oxidized lipid molecules have been shown to play a role in the regulation of immune responses.^1^ However, lipid oxidation products are more commonly associated with disrupting natural cellular processes, and contribute to aging and diseases such as Parkinson's, Alzheimer's, atherosclerosis, and cancer.^2^, ^3^

In the limiting case, extreme oxidation of cellular components can lead to cell death through apoptosis or necrosis, and this effect is successfully used in photodynamic therapy (PDT), a type of cancer treatment.^4^ PDT is a light‐activated process where a ′photosensitizer′, a molecule that produces ROS upon excitation by an appropriate wavelength of light, is targeted to malignant cells and tissues. Locally produced ROS efficiently oxidize cellular components, leading to the death of targeted cells. Given their abundance in cells, lipids serve as primary targets for ROS during PDT and membrane oxidation is a key step leading to cell apoptosis.^5^, ^6^

Consequently, significant effort has been made to understand physicochemical changes in membranes under oxidative stress. In model membrane systems the appearance of oxidized lipids was reported to increase the membrane surface area, causing spontaneous fluctuations of the membrane^7^, ^8^ and alterations in membrane curvature,^9^ permeability,^10^ and packing order.^11^ Lipid oxidation has also been shown to affect diffusion in model membranes.^9^, ^12^ However, the majority of the aforementioned effects have been observed in the bulk solution of model membranes (in large unilamellar vesicles, LUVs) lacking spatial resolution across the bilayer of an individual vesicle.

**Figure 1:**
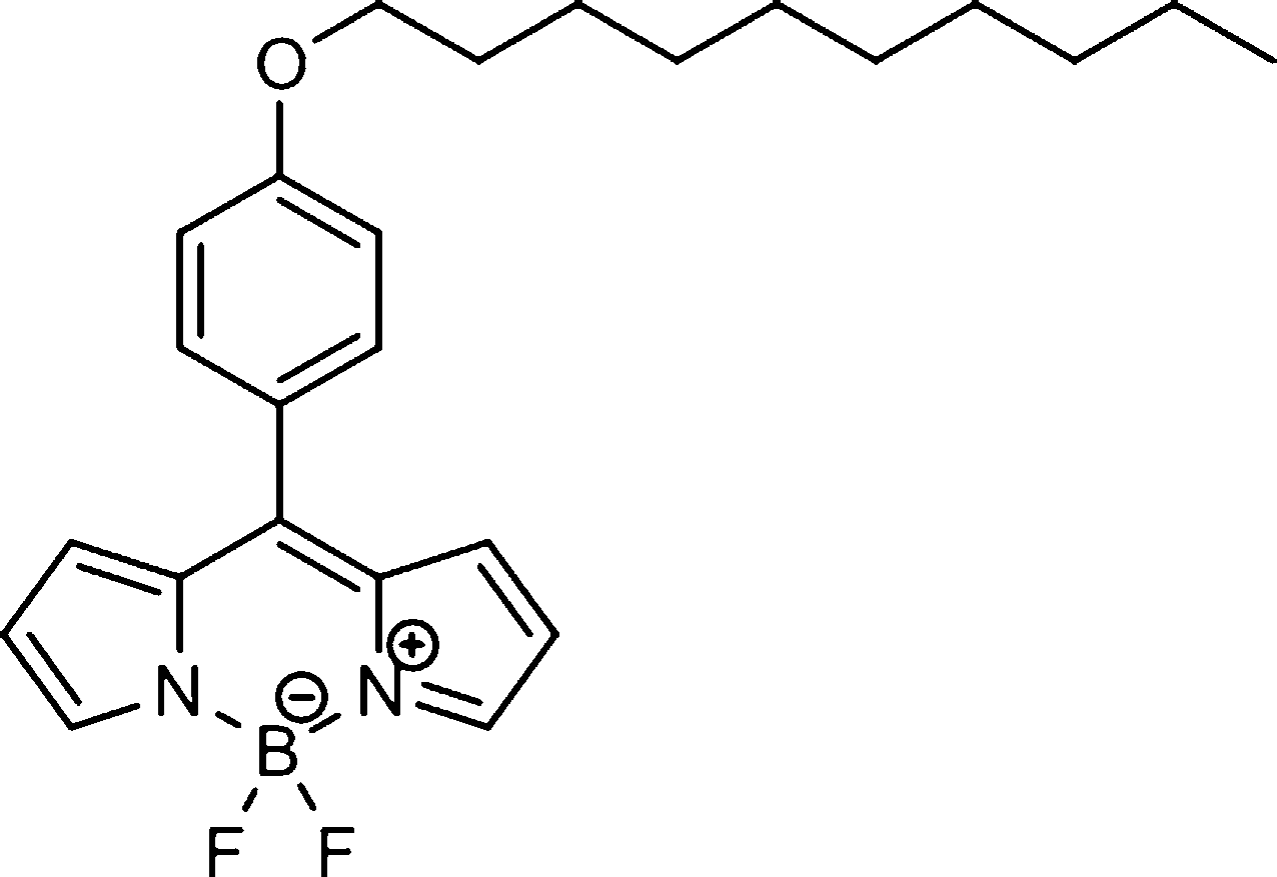
Molecular structures of BODIPY‐C_10_, used as a molecular rotor.

Fluorescence microscopy is a powerful tool for the visualization of lipid membranes. Consequently, a wide range of fluorescent probes suitable for probing multiple properties of lipid membranes was developed,^13^ including probes for sensing membrane potential and fluidity,^14^ for detecting lipid order in the outer lipid leaflet of the lipid bilayer^15^ and for sensing changes in the membrane during apoptosis.^16^ In this work, we utilized BODIPY‐C_10_ 17, 18 (Figure 1), a fluorophore that belongs to a group of dyes termed ‘molecular rotors’ that have viscosity‐dependent fluorescence quantum yields, lifetimes,^19^, ^20^ and depolarization.^21^, ^22^ When combined with fluorescence lifetime imaging microscopy (FLIM), molecular rotors can be used to obtain spatially resolved viscosity maps of microscopic objects,^17^, ^23^, ^24^, ^25^, ^26^, ^27^, ^28^, ^29^, ^30^, ^31^, ^32^, ^33^, ^34^, ^35^, ^36^, ^37^, ^38^, ^39^, ^40^ as well as to observe dynamic change in viscosity during relevant processes of interest.^37^, ^39^, ^41^, ^42^ Thus, we aimed to use BODIPY‐C_10_, which is known to completely embed into the fluid‐phase lipid bilayers^40^ to directly examine how photooxidation during PDT affects viscoelastic properties of model lipid membranes, with spatial‐ and time‐resolution. As a model system, we have employed giant unilamellar vesicles (GUVs) composed of an unsaturated lipid 1,2‐dioleoyl‐*sn*‐glycero‐3‐phosphocholine (DOPC), which is susceptible to oxidation by a variety of ROS. By imaging GUVs we are able to monitor the effects of oxidation away from an initially irradiated bilayer site, providing information on the nature of ROS involved in viscosity change and the mechanism of its action.

There are two distinct pathways for a photosensitizer to create ROS, Types I and II.^43^ The reaction diagrams of the resulting species with lipids are shown in Figure S1 in the Supporting Information.

In a Type II process, a triplet‐state photosensitizer can transfer its energy to a ground‐state oxygen molecule, producing singlet oxygen, ^1^O_2_. ^1^O_2_ is an oxidant, which is known to react with unsaturated lipids, producing peroxidation products.^7^ On the other hand, in a Type I reaction, a triplet‐state photosensitizer can act as an electron donor or can abstract hydrogen from surrounding molecules, creating radicals. Thus, a Type I oxidation process does not stop at a peroxidation stage, and the reaction proceeds further until the lipid molecule is cleaved along the double bond.^43^

We have selected a range of photosensitizers that participate in either Type I or Type II chemistry. Their structures are shown in Figure S2 in the Supporting Information. Firstly, we have used three different porphyrin‐based photosensitizers that are known to result in Type II oxidation by singlet oxygen, but occupy different positions relative to the hydrophobic core of a lipid bilayer. Namely, 1) hydrophobic tetraphenylporphyrin (TPP) will reside in the tail region of the lipid membrane; 2) hydrophilic tetrakis(4‐sulfonatophenyl)porphine (TPPS^4−^) will reside in the aqueous solution on the outside of the bilayer; and 3) a porphyrin dimer (PD), which was previously demonstrated to attach to the surface of the lipid bilayer.^39^ Secondly, we used methylene blue (MB) as a photosensitizer, which is known to participate in Type I reactions.^44^

Here we demonstrate that FLIM of lipid bilayers containing molecular rotor BODIPY‐C_10_ is a powerful tool for studying change in viscoelastic properties of membranes during oxidation. We examine how the localization of photosensitizer affects the bilayer's viscosity. Finally, we show a clear difference in the evolution of the membrane's viscoelastic properties during Type I and Type II photooxidation.

## Results and Discussion

We have prepared a series of DOPC GUVs that contained BODIPY‐C_10_, our molecular rotor, and various photosensitizers. We made sure that the viscosity‐sensitive fluorescence signal of BODIPY‐C_10_, (recorded between 510–600 nm) can be clearly separated from the fluorescence of photosensitizers. The absorption spectra of all dyes used in this study are given in Figure S3 in the Supporting Information. PD alone absorbs at the excitation wavelength of BODIPY‐C_10_ (480 nm). However, even though PD absorbs at 480 nm, its fluorescence is centered at 630–750 nm,^39^ which does not overlap with fluorescence of BODIPY‐C_10_ (510–600 nm). Thus, we made sure that the time‐resolved fluorescence decays recorded belong to the molecular rotor and are not contaminated by the signal from the PDT photosensitizer used. We note that all the photosensitizers can be individually excited using internal microscope laser wavelengths as shown in Figure S3 in the Supporting Information.

### Type II photooxidation

**Figure 2:**
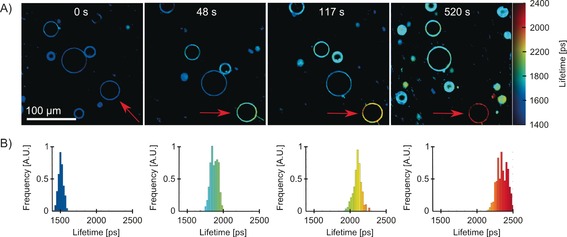
A) FLIM of BODIPY‐C_10_/PD DOPC GUVs during irradiation. Single GUV, which is shown by the red arrow, was irradiated at 453 nm by zooming in 5 times, for periods of time shown in each image. B) Fluorescence lifetime histograms of BODIPY‐C_10_ in the irradiated vesicle obtained upon 480 nm excitation. The lifetime increases from 1509±26 to 2254±53 ps corresponds to viscosity increase from 170±5 to 332±14 cP.

The first set of GUVs studied contained PD, a known singlet oxygen photosensitizer^45^, ^46^ that is bound to the lipid bilayer surface^39^ and BODIPY‐C_10_, which probes viscosity of the inner part of the lipid bilayer.^40^ Previously, we have utilized PD as both the photosensitizer and as a molecular rotor and recorded a large increase in the DOPC monolayer viscosity upon PDT.^39^ Here, we aimed to separate out the dual function of PD as a photosensitizer and a molecular rotor. By doing so we aimed to test whether: 1) the viscosity increase can be observed independently of the rotor used and its positioning in the bilayer and 2) whether ROS can penetrate the bilayer from the surface of the membrane when produced by an externally bound photosensitizer and can cause a viscosity increase within the bilayer, as probed by BODIPY‐C_10_. The results are presented in Figure 2. We have selected a single GUV (shown by the red arrow) by zooming in and irradiated it at 453 nm, where PD absorbs. Throughout irradiation we acquired several FLIM images of BODIPY‐C_10_. It is clear to see that progressive irradiation caused a continuous increase in fluorescence lifetime from 1509±26 to 2254±53 ps, corresponding to a viscosity increase from 170±5 to 332±14 cP (Figure 2 B).

Furthermore, we performed three control experiments (see Figure S4 in the Supporting Information). First, we prepared DOPC GUVs containing BODIPY‐C_10_ only, without PD, and irradiated it at 453 nm. Secondly, we prepared GUVs using DPhPC, a saturated lipid that does not contain double bonds, making the bilayer unreactive to ROS. Finally, we tested the irradiation effects in the presence of 0.11 m NaN_3_, an efficient singlet oxygen quencher. In all three control experiments no change in fluorescence lifetime of BODIPY‐C_10_ was observed during irradiation (Figure S4 in the Supporting Information). This data allowed us to conclude that, for the viscosity change seen in Figure 2 to take place, both singlet oxygen and unsaturated bonds are required; therefore, this change is likely caused by the oxidation of unsaturated bonds in lipid molecules by singlet oxygen.

**Figure 3:**
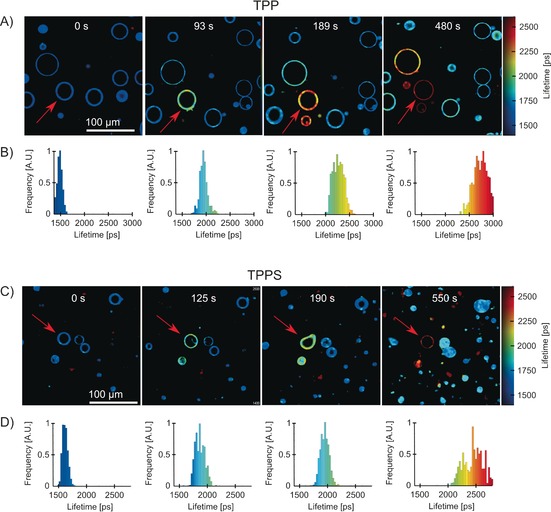
FLIM of DOPC GUVs containing BODIPY‐C_10_ as a viscosity probe and either TPP (A, B) or TPPS^4−^ (C, D) as photosensitizers during irradiation at 420 nm. TPP was embedded in the lipid bilayer at 1:1000 lipid‐to‐dye ratio, whereas TPPS^4−^ was directly dissolved in water (10 μm). The irradiated GUVs are shown by red arrows; irradiation times are shown above the images. The lifetime distributions of irradiated vesicles (B, D) are shown in the panels below each image. During irradiation, some vesicles leaked lipid material or underwent deformation before returning back to a spherical shape following further irradiation. The lifetime of BODIPY‐C_10_ increased from 1544±49 to 2737±138 ps (176±8 to 482±48 cP) using TPP and from 1598±38 to 2486±190 ps (186±7 to 399±55 cP) using TPPS^4−^.

It was suggested previously that singlet oxygen reacts with double bonds in an ‘ene’ type reaction, which leads to the insertion of a hydroperoxide group next to a double bond in lipid molecules.^7^, ^43^ Such a change is likely to force the reacted lipid molecule to curve in order to insert the formed hydrophilic hydroperoxide group into the aqueous phase.^7^ We hypothesize that this change leads to an increase of microviscosity in the hydrophobic core of the membrane where BODIPY‐C_10_ resides. We stress the fact that the lifetime changes gradually in the whole vesicle, which means that microviscosity in the GUV increases gradually with the increasing amount of the oxidized lipid molecules without any visible phase separation. The change is accompanied by the loss of lipid material from the vesicle, which was observed during the imaging. Examples of such behavior can be seen in Figure 2 A after 48, 127, and 520 s of irradiation, and in Figure 3 C after 125 s of irradiation, where the irradiated vesicles show thin structures extending away from the lipid shell. The loss of lipid material is consistent with data reported previously.^47^, ^48^, ^49^ We note that the fluorescence lifetimes of other DOPC vesicles in the large field of view (Figure 2) increase slightly as well, even though they were not directly irradiated. This could be due either to oxidation during FLIM imaging of BODIPY‐C_10_ or by longer‐lived ROS, which diffused away from the initially irradiated region.

We next set out to investigate if the dynamics of microviscosity increase can be affected by the location of the photosensitizer relative to the rotor within the bilayer, Figure 3. We used a hydrophobic porphyrin, TPP, which, due to its hydrophobic structure and neutral charge, is expected to be fully embedded in the hydrophobic core of the lipid bilayer. Next, a water soluble porphyrin, TPPS^4−^, was used, which readily dissolves in an aqueous solution and does not strongly interact with the lipid bilayer, as confirmed by fluorescence imaging (Figure S5 in the Supporting Information).

Upon irradiation of individual vesicles (Figure 3), in both cases the lifetime of BODIPY‐C_10_ increased gradually, in a similar manner that shown in Figure 2 with PD, from 1550–1600 to 2500–2750 ps, corresponding to viscosity change from 180 to 400–500 cP. Throughout the oxidation, vesicles leaked lipid material and went through a stage of rapid fluctuations (Figure 3), similar to what was observed using PD as a sensitizer. When the irradiation was paused during a shape fluctuation, the majority of vesicles retained their deformed shape (e.g., Figure 3 b at 190 s). This deformed shape did not produce inhomogeneous viscosity distribution across the vesicle. Surprisingly, the GUV returned to its spherical shape following further irradiation. We hypothesize that these temporary changes in shape are due to changes in the curvature of the bilayer induced by the presence of oxidized lipids. However, these are then released by excess lipid shedding during further irradiation.

Taken together, the results recorded in the presence of three singlet‐oxygen photosensitizers seem to indicate that the membrane is efficiently oxidized by singlet oxygen, irrespective of whether it was produced inside, outside, or on the surface of the bilayer. We would like to point out the fact that, due to a short lifetime of ^1^O_2_ in water (3.5 μs),^50^ it has limited time to diffuse into the membrane unless sensitized in close proximity to the bilayer, by the water‐soluble photosensitizer TPPS^4−^.

**Figure 4:**
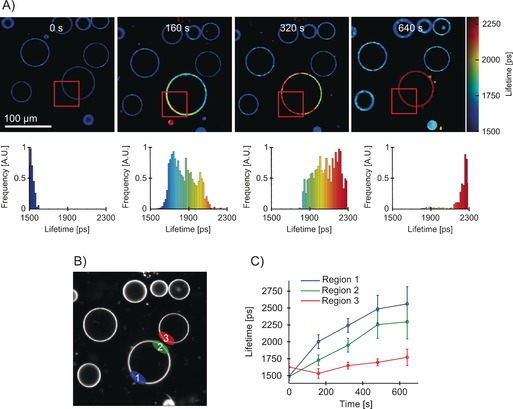
FLIM of a partially irradiated DOPC GUVs containing BODIPY‐C_10_ as a viscosity probe and TPP as a photosensitiser. A) The irradiation at 420 nm was performed for the lengths of times given in each image across the red rectangular area highlighted. The lifetime histograms of the irradiated GUV are shown on the bottom. TPP was embedded in the lipid bilayer at 1:1000 lipid‐to‐dye ratio. B) Regions of interest over which the fluorescence decays were analyzed, as shown in (C). C) Fluorescence lifetime evolution recorded in various GUV regions during irradiation of a part of GUV. The error bars are equal to one standard deviation. The lifetime change was highest in the irradiated section of the vesicle, Region 1 (ca. 1500 to ca. 2500 ps). The change was smaller in the non‐irradiated section of the same vesicle (ca. 1500 to ca. 2250 ps). No change was observed in the adjacent non‐irradiated vesicle, Region 3.

Next, we set out to examine the mobility of oxidized lipids within a single GUV. We irradiated part of a single DOPC vesicle and followed a change in lifetime of BODIPY‐C_10_ in the whole vesicle using FLIM (Figure 4). TPP was chosen as a photosensitizer because it embeds in the hydrophobic part of the lipid bilayer and is not present in the aqueous solution outside the bilayer.

It is clear to see that the irradiated section of GUV (showed by the red rectangle in Figure 4) has an increased lifetime compared to the rest of the vesicle and the rest of the image. The fluorescence lifetime of BODIPY‐C_10_ in the irradiated section of the vesicle (Figure 4, Region 1) increased faster during irradiation, compared to the value in the non‐irradiated part in the same GUV (Region 2). Both these values were considerably higher than the lifetime in a non‐irradiated GUV (Region 3) adjacent to the irradiated vesicle. Though the lifetime increase was the highest in the directly irradiated area (ca. 1500 to ca. 2500 ps, equivalent to 170 to 400 cP), we observed that the lifetime of BODIPY‐C_10_, indicative of microviscosity, gradually increased in the whole vesicle. For example, the non‐irradiated part of the same vesicle (Region 2) showed an increase from ≈1500 to ≈2250 ps, equivalent to 170 to 330 cP. At the same time, an adjacent vesicle (Region 3) showed almost no lifetime change at all. This lack of change in Region 3 must indicate that, as expected, the ROS responsible for microviscosity increase in the DOPC bilayer can efficiently travel through the bilayer (to Region 2), but cannot travel long distances in an aqueous solution.

Although single oxygen is the main ROS produced by the Type II photosensitizer TPP, we note that, given the known lifetime of ^1^O_2_ in the DOPC lipid bilayers of ≈35 μs,^51^ it is only expected to travel approximately 3 μm distance within three times its lifetime.^5^ An alternative explanation is that the ROS initially produced cause lipid peroxidation at the point of irradiation and then the lipid oxidation products travel within the same vesicle (but not in an aqueous solution), leading to a gradual viscosity increase in the whole vesicle.

**Figure 5:**
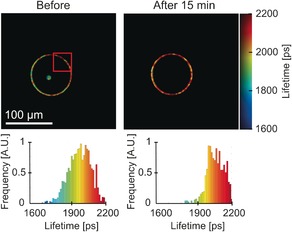
FLIM images of a partially irradiated DOPC GUV incorporating BODIPY‐C_10_ as a viscosity probe and TPP as a photosensitizer. The part of the vesicle highlighted by the red rectangle was irradiated at 420 nm for 1 min. The FLIM images of the same vesicle immediately after the irradiation (left) and 15 min after the irradiation was completed (right) are shown along with the corresponding lifetime histograms below each image.

To distinguish between these two scenarios, we partially irradiated a GUV (see Figure 5) for 1 min, to achieve a contrast in viscosity between the irradiated and the non‐irradiated part (equivalent to 125 ps lifetime difference). We consequently recorded a FLIM image of the same vesicle 15 min later, to see if the viscosity variations within the vesicle remained after the irradiation was complete. The premise here was that most of the short‐lived ROS, and in particular singlet oxygen, will decay and/or react during the irradiation period only, and will not be able to cause further oxidation after the irradiation was completed. The oxidized lipids, on the other hand, should be able to diffuse across the whole vesicle even after the irradiation has been completed.

Our data (Figure 5) show that, immediately following irradiation, the fluorescence lifetime of BODIPY‐C_10_ in the irradiated and the non‐irradiated parts were 1950 and 2075 ps, respectively, corresponding to viscosities of 260 and 290 cP, whereas in the image recorded following a 15 min delay, we did not observe any variations in the viscosity across the vesicle, giving the overall lifetime of ≈2100 ps. This data appears to indicate that diffusion of the lipid peroxidation product contributes to the observed processes, because singlet oxygen is not able to survive for 15 min and cause the observed changes. On the other hand, we note that the viscosity across the vesicle did not simply stagnate between 260 and 290 cP; instead, the non‐irradiated region displayed a viscosity increase, producing the high final viscosity of 290 cP in the whole vesicle. This result suggests that a relatively long‐lived oxidizing species is present in the system and is able to diffuse within the vesicle, causing further oxidation even after the irradiation was complete. One possibility is that lipid peroxides that diffuse across the vesicle can slowly decompose, leading to further ROS formation that oxidize other lipids in the vicinity. We note that the fluctuations of the vesicle shape observed in the course of our experiments must assist in lipid mixing and diffusion within the continuous bilayer length.

### Type I photooxidation

As previously mentioned, Type I photosensitization involving electron transfer or hydrogen abstraction, can occur from the photosensitizer triplet state.^43^ In the previous section we utilized porphyrin‐based photosensitizers characterized by high singlet‐oxygen yields that were likely to participate in Type II photosensitization. Here, we compare this with microviscosity evolution in the presence of methylene blue (MB), a photooxidant with Type I properties.^44^

**Figure 6:**
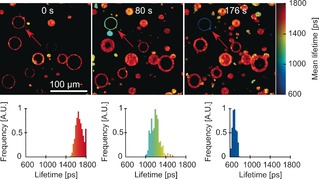
FLIM of DOPC GUVs containing BODIPY‐C_10_ as a viscosity probe and a water‐soluble MB as photosensitizer (10 μm). The GUV irradiated at 633 nm is shown by the red arrow; irradiation times are shown above the images. The distributions of intensity‐weighted mean lifetimes in an irradiated GUV are shown in the panels below each image.

The results of 633 nm irradiation of a single vesicle in the presence of MB in the incubation medium are shown in Figure 6. The observed evolution of the BODIPY‐C_10_ fluorescence lifetime was surprising and strongly contrasted the results obtained with porphyrin‐based photosensitizers. During irradiation of a single vesicle with MB present in the incubation solution, the mean fluorescence lifetime of BODIPY‐C_10_ decreased rather than increased, as we observed with Type II photosensitizers (Figure 6). Furthermore, the BODIPY‐C_10_ fluorescence decays in the irradiated vesicles were best‐fitted with a biexponential function (see Figure S6 in the Supporting Information).

BODIPY‐C_10_ is characterized by monoexponential fluorescence decays, if recorded in homogeneous medium and in the absence of the aggregates.^17^ Hence, the biexponential decays observed during Type I peroxidation can be either the result of two kinds of lipid environments created by peroxidation or the presence of aggregates.^17^ We first tested if biexponential decays of BODIPY‐C_10_ are the result of the aggregation of BODIPY‐C_10_. The aggregates are known to cause quenching of the BODIPY‐C_10_ monomeric species, with the long‐lived fluorescence of the aggregates appearing in the red region of the spectrum (>570 nm), overall leading to a biexponential decay.^17^ Hence, we recorded the fluorescence decay traces over two detection windows, 500–550 and 600–650 nm (Figure S6 in the Supporting Information). We observed no difference between the two traces recorded over these different detection ranges, which ruled out the presence of aggregates in the oxidized GUVs.

We have also tested if the biexponential decay was caused by oxidation of BODIPY‐C_10_ itself by MB. For this, we repeated the oxidation experiment using GUVs made out of saturated DPhPC lipid, which is resistant to oxidation by ROS. The DPhPC GUVs showed no change in lifetime upon irradiation, confirming that the BODIPY‐C_10_ itself was not affected by the ROS produced (Figure S7 in the Supporting Information). It should be noted that, in all experiments involving MB as a photooxidant, bleaching of BODIPY‐C_10_ was observed. However, the constant lifetime observed in the DPhPC control experiment confirms that the bleaching does not affect the validity of FLIM data.

Thus, the presence of the two lifetime components in the fluorescence decays recorded during Type I oxidation of DOPC GUVs indicate the presence of two environments in the lipid bilayer, as sensed by the molecular rotor. Again, we did not detect any large‐scale phase separation in the oxidized vesicle, which means that the domains that form were small and below our resolution limit. Alternatively, it is possible that, in the bilayer produced by Type I oxidation, BODIPY‐C_10_ is able to adopt two positions, similar to what was previously detected in gel‐phase bilayers constructed from 1,2‐dipalmitoyl‐*sn*‐glycero‐3‐phosphocholine (DPPC) or sphingomyelin.^40^ The fluorescence lifetimes extracted from the biexponential fitting of oxidized GUVs were both decreasing during the irradiation (Figure S8 in the Supporting Information), indicating the decrease in the effective viscosity sensed by BODIPY‐C_10_.

This intriguing decrease in viscosity observed during Type I lipid photooxidation can be rationalized based on the literature data. It is known that Type I oxidation results in a cleavage through a double bond in lipid molecules,^43^, ^47^, ^48^ which in turn could lead to a very loose packing of lipids and increased volume for BODIPY's intramolecular rotation. Pore formation^43^, ^47^, ^48^ and a higher fluidity of the membrane^12^ were previously reported as a result of Type I oxidation, which agree with our findings using direct viscosity measurements with the molecular rotor.

Finally, we performed oxidation experiment of DOPC GUVs, with MB as a photosensitizer in the presence of NaN_3_ as a singlet‐oxygen quencher (Figure S9 in the Supporting Information). Here, we also observed biexponential fluorescence decays of BODIPY‐C_10_ following irradiation, and saw a decrease in mean fluorescence lifetime. However, the lifetime change observed in the presence of NaN_3_ was significantly slower, which was consistent with MB being mixed Type I and Type II oxidant. NaN_3_ stops Type II oxidation path, which must otherwise assist Type I oxidation through peroxide formation. Nevertheless, a pure Type I oxidation (through peroxide formation followed by lipid cleavage)^22^ is able to proceed, which led to a decrease of BODIPY‐C_10_ lifetime, as in the absence of NaN_3_.

## Conclusion

In conclusion, we have utilized a hydrophobic molecular rotor that was fully embedded in a lipid bilayer to probe the effect of photooxidation on the bilayer viscosity. We established that Type II photooxidation of unsaturated lipid bilayers causes a large increase of bilayer microviscosity, irrespective of the relative position of the photosensitizer used to produce singlet oxygen: outside, within, or on the surface of the bilayer. By investigating the evolution of viscosity in partially irradiated vesicles, we concluded that the viscosity change is likely caused by the diffusion of lipid peroxides within the bilayer. In contrast to Type II oxidation, we have detected a large decrease in viscosity during Type I photooxidation using methylene blue as a photosensitizer. Thus, molecular rotor BODIPY‐C_10_ can clearly discriminate between the microviscosity changes during Type I and Type II chemistry. Overall, our results demonstrate that viscosity sensor BODIPY‐C_10_ is a very useful tool for investigating photooxidation of model lipid membranes, which provides spatial and temporal resolution unavailable previously.

## Experimental Section

### Materials

PD was synthesized as described^52^ previously and kindly provided by the group of Prof H.L. Anderson. BODIPY‐C_10_ was synthesized by the method previously reported.^53^ TPP was obtained from Aldrich (97 % purity), TPPS^4−^ was obtained from Sigma–Aldrich, and MB was obtained from Hopkin and Williams. Stock solutions of 1,2‐dioleoyl‐*sn*‐glycero‐3‐phosphocholine (DOPC) and 1,2‐diphytanoyl‐*sn*‐glycero‐3‐phosphocholine (DPhPC) in chloroform were obtained from Avanti Polar Lipids. All solvents used were of spectroscopic grade.

### Preparation of giant unilamellar vesicles (GUVs)

GUVs were prepared by electroformation. A 5 μL of mixture of lipid (2 mg mL^−1^) was spread on ITO (indium–tin oxide) slide on 1 cm^2^ area. Then the solution was evaporated under 2 MPa pressure for 30 min. The electroformation chamber was then assembled out of two ITO slides with polydimethylsiloxane (PDMS) spacer in between. The chamber was filled with 200 mm sucrose solution in water, connected to a TTi TG550 function generator and 1.2 V voltage at 10 Hz frequency was applied for 1.5 h. The chamber was kept incubated at 60 °C during electroformation. The newly formed GUVs were transferred into aqueous 200 mm glucose solution in Lab‐Tek chamber slides for imaging. For appropriate experiments, water‐soluble photosensitizers (TPPS^4−^, MB) were dissolved in the mixture used for imaging at 10 μm concentration. Other photosensitizers (PD, TPP) were incorporated by mixing them with lipids in chloroform before electroformation at 1000:1 lipid‐to‐dye ratio. BODIPY‐C_10_ was either included in the mixture with lipids at 300:1 lipid‐to‐dye ratio or 10 μL solution of BODIPY‐C_10_ in methanol (50 μm) was added into 400 μL of the mixture used for imaging. The fluorescence lifetime of BODIPY‐C_10_ matched that previously reported for DOPC bilayers, independent of the preparation method.^40^

### Acquisition of fluorescence lifetime images

FLIM images were recorded using the Leica SPII confocal laser‐scanning microscope together with a Coherent Chameleon Vision II mode‐locked femtosecond Ti:sapphire laser and a Becker & Hickl SPC‐830 (time‐correlated single‐photon counting) TCSPC card. The pulse length and pulsing frequency was 140 fs and 80 MHz. The output wavelength was tuneable between 680 and 1080 nm. The required laser wavelength was obtained by frequency doubling the output of the Ti:sapphire laser with second harmonic generation crystal (SHG, Harmonic, Coherent). BODIPY‐C_10_ was excited at 480 nm. Photosensitizer excitation wavelengths were 453 nm for PD, 420 nm for TPP and TPPS^4−^, and 633 nm for MB. An internal HeNe laser was used for 633 nm excitation. The BODIPY‐C_10_ lifetime for FLIM was detected between 510 and 600 nm using a photomultiplier tube (PMC‐100‐1, Hamamatsu) with a x63 water immersion objective, the confocal pinhole was half‐open at 300 μm, which is equivalent to 2.7 Airy units. The optimal pinhole size of 1 Airy unit for maximizing axial resolution was not used because it results in smaller fluorescence collection efficiency and the high axial resolution was not required for imaging vesicles larger than 10 μm in diameter. The irradiation of photosensitizers in part of the image to achieve PDT was performed by zooming in 4–6 times and by continuous scanning across the zoomed area for a length of time indicated. FLIM images were acquired at 256×256 pixel resolution using 256 time bins. The scanning frequency was 400 Hz. The instrument response function (IRF) was obtained by recording the scattering curve from a glass coverslip.

### Data analysis

FLIM images were fitted and analyzed using the FLIMfit software tool developed at Imperial College London (v4.6.1).^54^ 5×5 pixel binning was needed in order to get >100 counts at the peak of each decay. Data processing and analysis was done in MATLAB R2012a and OriginPro 8.6. The lifetime values were converted to viscosities using the calibration curve recorded earlier.^17^

## Supporting information

As a service to our authors and readers, this journal provides supporting information supplied by the authors. Such materials are peer reviewed and may be re‐organized for online delivery, but are not copy‐edited or typeset. Technical support issues arising from supporting information (other than missing files) should be addressed to the authors.

Supplementary
