# Supplementary material for: A Molecular Rotor that Measures Dynamic Changes of Lipid Bilayer Viscosity Caused by Oxidative Stress
Source: Chemistry. 2016 Aug 3;22(37):13210–7. doi: 10.1002/chem.201601925 (PMC5096028; doi:10.1002/chem.201601925)
Supplement: Supplementary file 1 — Supplementary [file CHEM-22-13210-s001.pdf]

CHEMISTRY
A European Journal

Supporting Information

A Molecular Rotor that Measures Dynamic Changes of Lipid Bilayer Viscosity Caused by Oxidative Stress

Aurimas Vyšniauskas, Maryam Qurashi, and Marina K. Kuimova*[a]

chem_201601925_sm_miscellaneous_information.pdf

Supporting Information

Table of Contents

Additional data S2

Figure S1 .... S2

Figure S2 .... S3

Figure S3 .... S4

Figure S4 .... S5

Figure S5 .... S6

Figure S6 .... S7

Figure S7 .... S8

Figure S8 .... S8

Figure S9 .... S9

Bibliography S9

1. Additional data

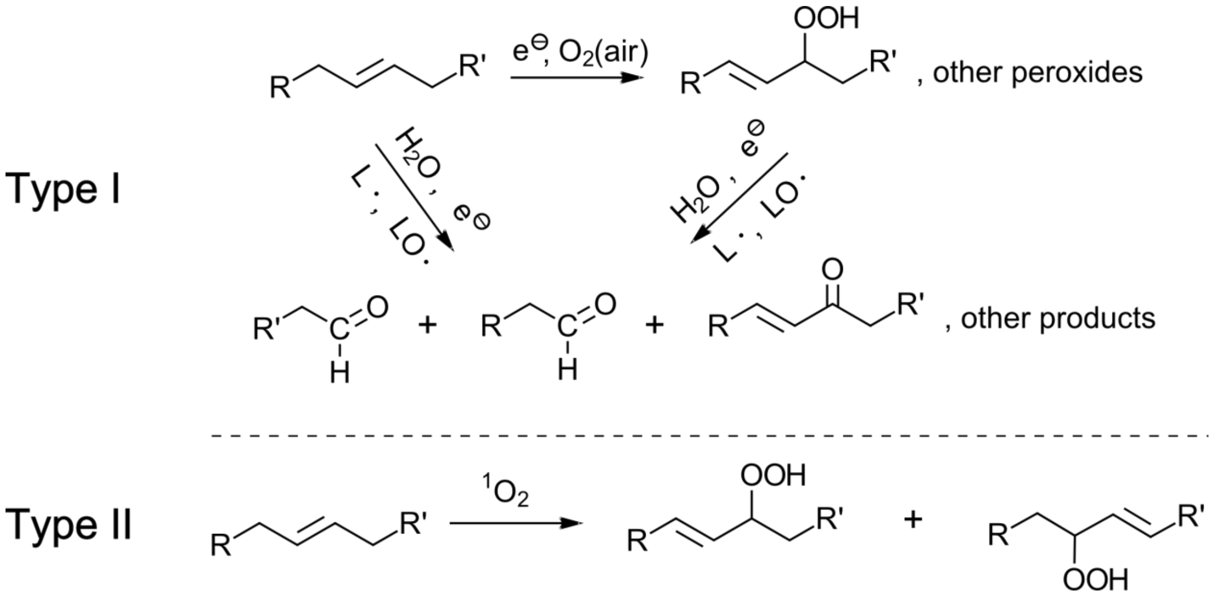

Figure S1. The reaction diagrams for Type I and Type II lipid oxidations of unsaturated lipids.[1] Type I oxidation is caused by an excited photosensitiser, which transfers its electron to the surrounding molecules and starts radical chain reactions leading to lipid peroxides, ketones aldehydes and double bond cleavage. **R** and **R’** denote the rest of the unsaturated lipid molecule around its double bond, **L·** and **LO·** are lipid radicals, which are formed as intermediates during Type I oxidation. Type II oxidation is caused by photosensitiser, which excites oxygen to its singlet state, which then reacts with unsaturated lipids leading to peroxides where the oxidation stops.

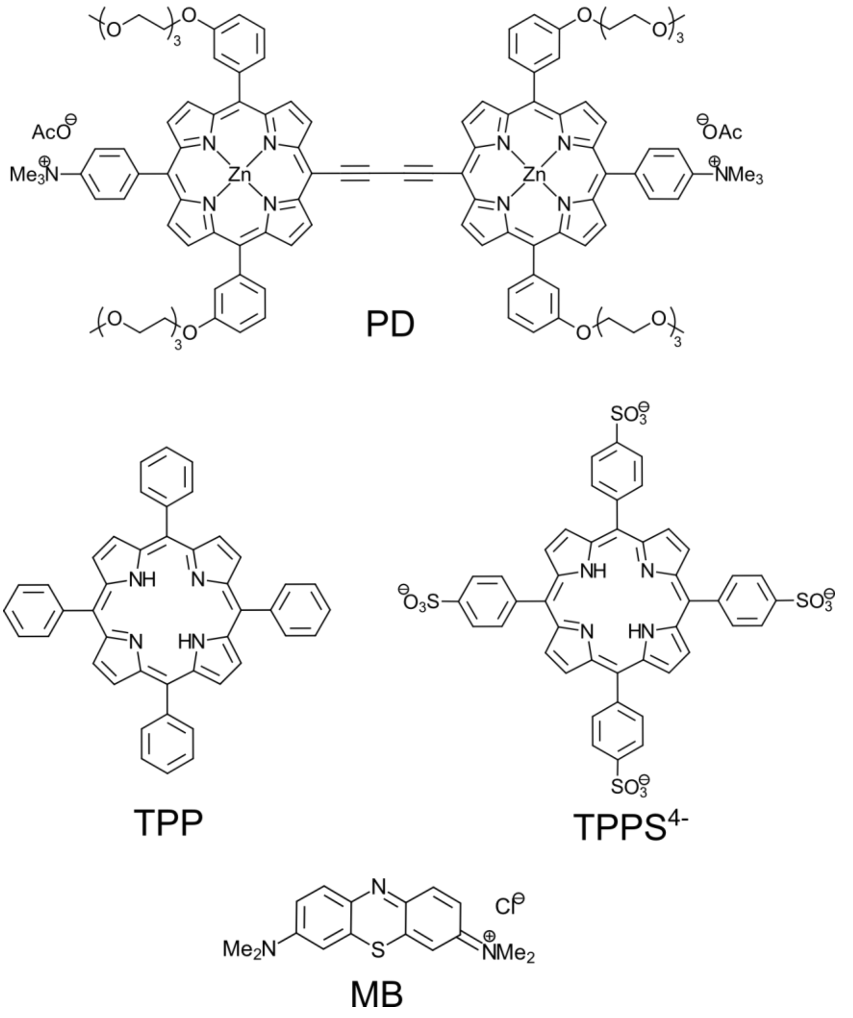

Figure S2. The structures of the photosensitisers used in this work, which were porphyrin dimer (PD), tetraphenylporphyrin (TPP), tetrakis(4-sulfonatophenyl)porphine ($TPPS^{4-}$) and methylene blue (MB).

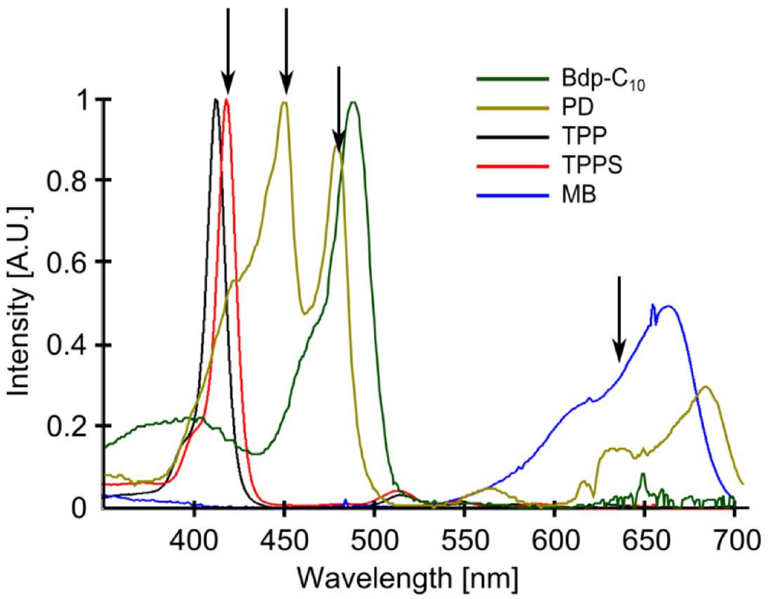

Figure S3. Absorption spectra of photosensitisers PD, TPP, TPPS and MB together with spectra of molecular rotor BODIPY-C_{10}. Excitation wavelengths for BODIPY-C_{10} (480 nm) and irradiation wavelengths for all photosensitisers (420, 453, 633 nm) are shown by the black arrows. Measured fluorescence of BODIPY-C_{10} cannot be contaminated by fluorescence of any photosensitiser because TPP, TPPs and MB do not absorb at 480 nm and PD fluoresces at a significantly longer wavelength (630-750 nm) compared to BODIPY-C_{10} (510-600 nm).

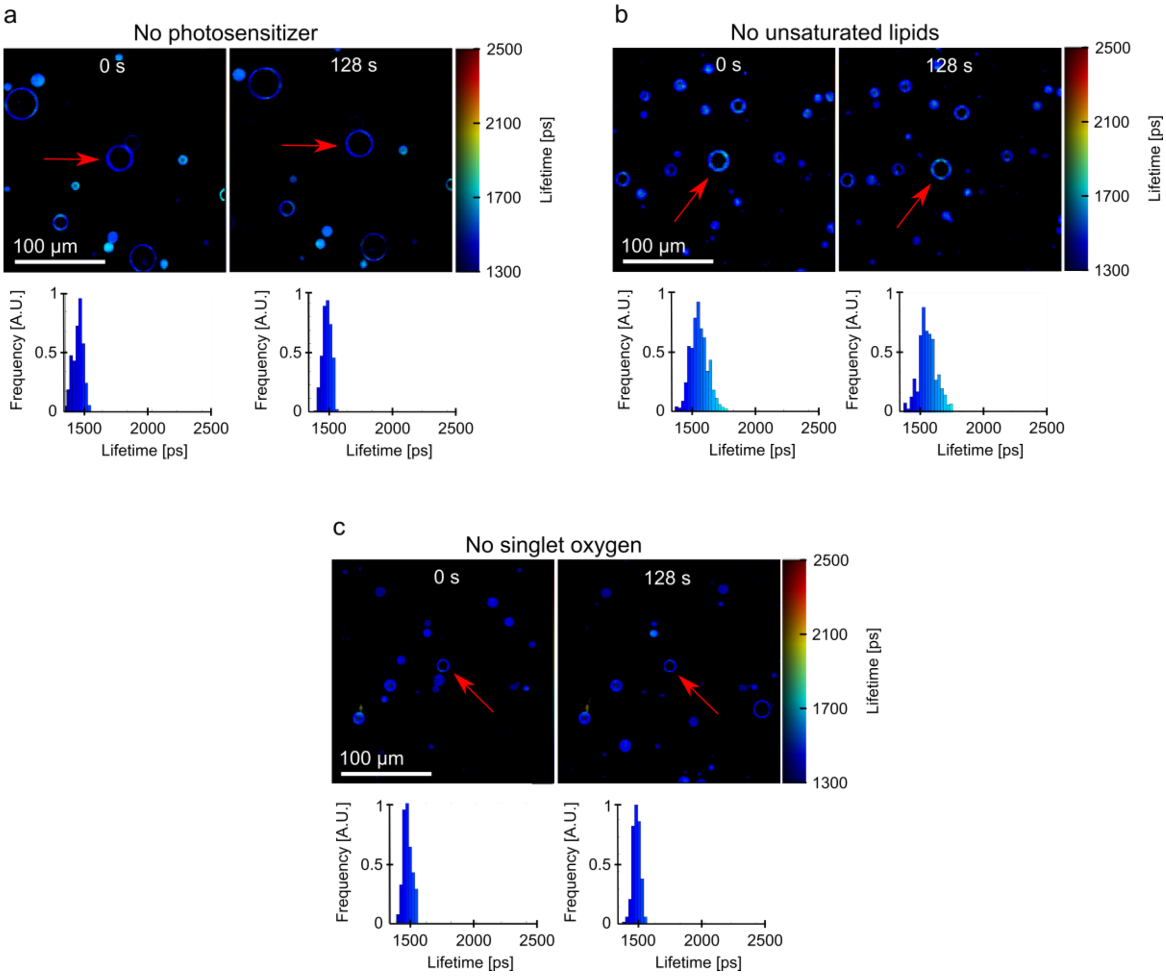

Figure S4. Control experiments for oxidation of GUVs using porphyrin dimer PD monitored by BODIPY-C_{10} (Figure 1, main text). FLIM images and corresponding lifetime histograms were recorded (a) when PD was absent in the DOPC/BODIPY-C_{10} GUV; (b) when the oxidation-resistant saturated lipid DPhPC was used; (c) in the presence of the singlet oxygen quencher NaN_3 at 0.11M . The irradiated GUVs are shown by red arrows. Irradiation times are shown at the top of the images. FLIM images do not show any change in lifetime of irradiated GUVs in these control experiments.

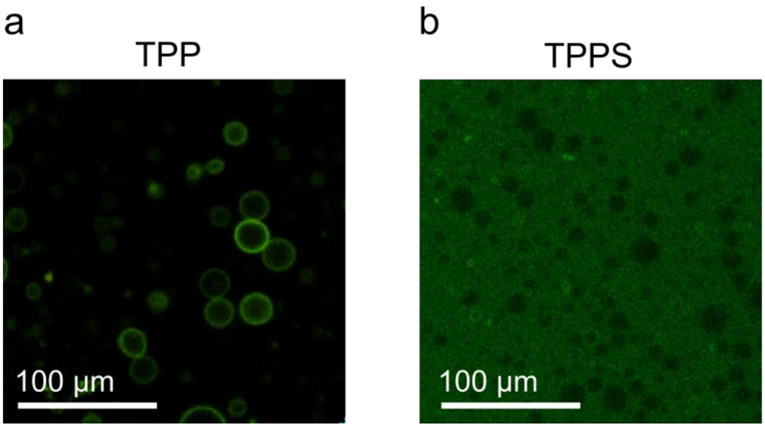

Figure S5. Confocal fluorescence images of DOPC GUVs with (a) TPP at lipid-to-dye ratio of 1:1000 and (b) with 10 µM of TPPS dissolved in the solution containing lipid- only GUVs. The images show that when present in the mixture with DOPC prior to GUV formation, TPP is localized in lipid membrane only, whereas $TPPS^{4-}$ does not incorporate into the bilayer from aqueous solution. The fluorescence was excited at 420 nm and detected at 600-700 nm.

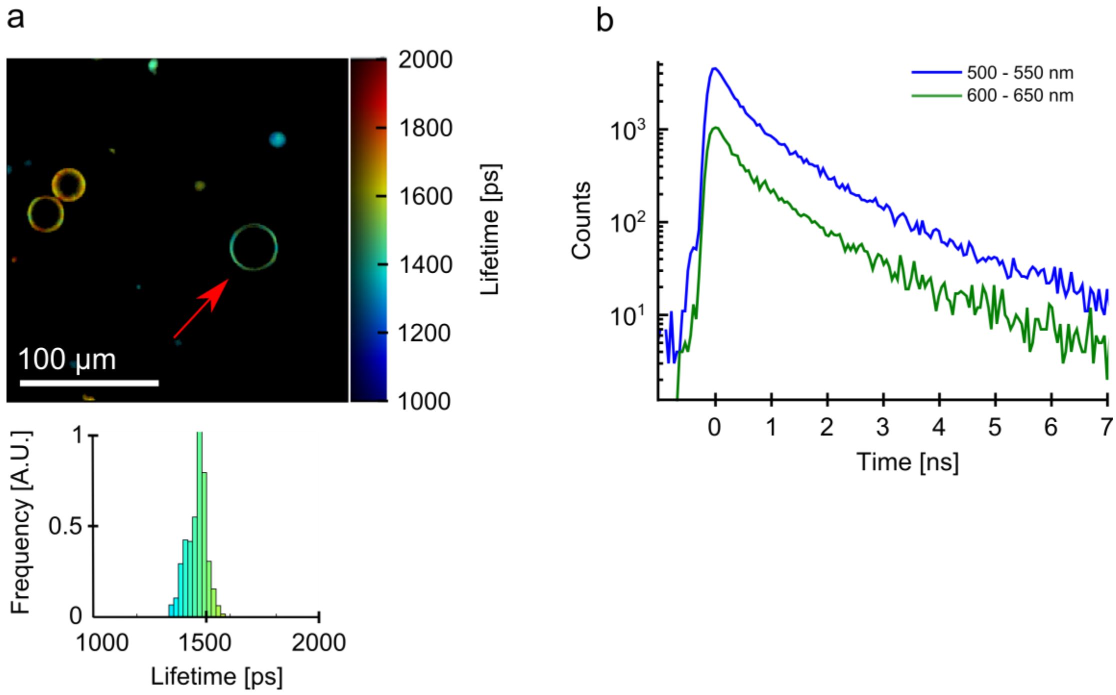

Figure S6. Control experiments verifying that BODIPY-C_{10} does not aggregate under experimental conditions corresponding to Type I lipid photooxidation.

Aggregation of BODIPY-C_{10} is known to result in fluorescent species emitting at 600-650 nm, which has a longer fluorescence lifetime compared to the non-aggregated dye[2]. Thus, the wavelength dependence of a lifetime signal can reveal the presence of the aggregated species.

a) FLIM image of BODIPY-C_{10} in DOPC GUVs in the presence of 10 μM of methylene blue in water before irradiation at 633 nm. The distribution of lifetimes within the GUV prior to irradiation is shown below the image.

b) Fluorescence decays of BODIPY-C_{10} in the GUV irradiated for 128 s at 633 nm. The decays collected at 500-550 nm (blue) and at 600-650 nm (green) are similar and indicate that BODIPY-C_{10} is not aggregated. The lifetimes of 500-550 nm decay are 1134 ps (23 % amplitude) and 218 ps (77%). The lifetimes of 600-650 nm decay are 1084 ps (22%) and 200 ps (78 %).

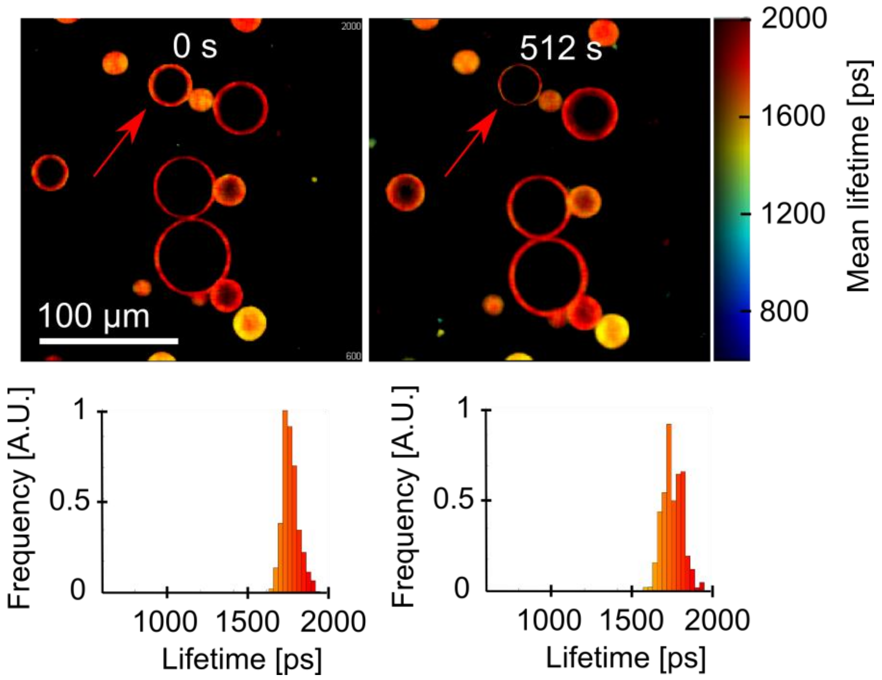

Figure S7. Photo-oxidation of DPhPC GUVs by methylene blue (Type I sensitiser).

When a fully saturated lipid DPhPC is used instead of DOPC we expect no oxidation to take place. In agreement with this, FLIM images of BODIPY-C_{10} in DPhPC in the presence of MB show no change in lifetimes of the irradiated GUV, which is marked by a red arrow. Irradiation time was long enough to cause bleaching of BODIPY-C_{10} but the lifetime was not affected.

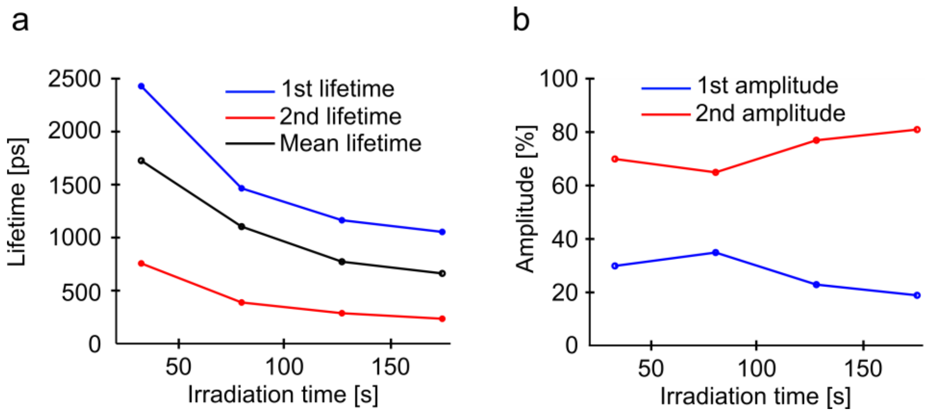

Figure S8. Biexponential analysis of FLIM images of BODIPY-C_{10} in an irradiated DOPC GUV with methylene blue present in the aqueous phase (Figure 5, main text). (a) Lifetimes and (b) amplitudes are mean values for the whole irradiated GUV. Lifetimes extracted from both exponential components decreased with irradiation time, indicating that two kinds of microenvironments for BODIPY-C_{10} were observed, which were becoming less viscous during irradiation.

Amplitudes of the two components indicate that most BODIPY-C_{10} molecules were in a less viscous environment, characterised by a shorter lifetime. Images were binned at 11x11 pixels in order to collect 1000 or more counts at the peak of the fluorescence decays to enable accurate biexponential fitting.

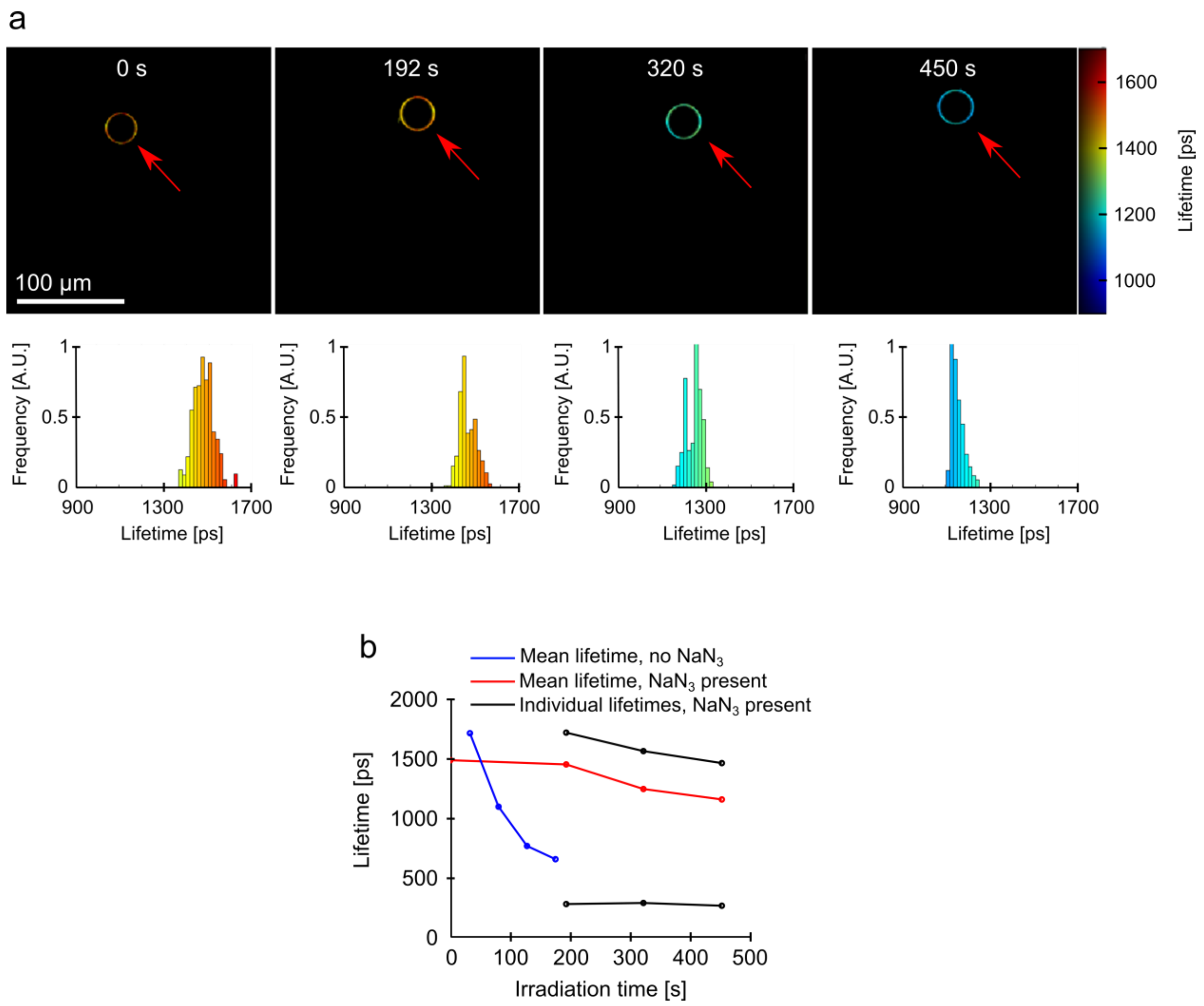

Figure S9. Oxidation of DOPC lipid GUVs by methylene blue in the presence of 0.11 M NaN_3 in the aqueous phase. a) A series of FLIM images of the GUV throughout irradiation at 633 nm. The decay of BODIPY-C_{10} is monoexponential at first (image at 0 s) but it becomes biexponential when the vesicle is irradiated. Following three images show the intensity-weighted mean lifetime of BODIPY-C_{10} during irradiation; corresponding histograms are shown below each image.

b) Intensity-weighted mean lifetime (red) and individual lifetimes (black) of BODIPY-C_{10} in the irradiated vesicle compared with lifetime trend obtained in the absence of NaN_3. In the presence of NaN_3 viscosity change reported by BODIPY-C_{10} is significantly slower but the trend remains the same.

References

1. Itri, R., Junqueira, H. C., Mertins, O. & Baptista, M. S. Membrane changes under oxidative stress: The impact of oxidized lipids. *Biophys. Rev.* **6,** 47–61 (2014).

2. Wu, Y. *et al.* Molecular rheometry: direct determination of viscosity in Lo and Ld lipid phases via fluorescence lifetime imaging. *Phys. Chem. Chem. Phys.* **15,** 14986–93 (2013).
